# Hybrid FDG PET/MRI vs. FDG PET and CT in patients with suspected dementia – A comparison of diagnostic yield and propagated influence on clinical diagnosis and patient management

**DOI:** 10.1371/journal.pone.0216409

**Published:** 2019-05-02

**Authors:** Nicolai Stefan Kaltoft, Lisbeth Marner, Vibeke Andree Larsen, Steen Gregers Hasselbalch, Ian Law, Otto Mølby Henriksen

**Affiliations:** 1 Department of Radiology, Rigshospitalet Blegdamsvej, Copenhagen University Hospital, Copenhagen, Denmark; 2 Department of Clinical Physiology, Nuclear Medicine and PET, Rigshospitalet Blegdamsvej, Copenhagen University Hospital, Copenhagen, Denmark; 3 Danish Dementia Research Centre, Dept. of Neurology, Rigshospitalet Blegdamsvej, Copenhagen University Hospital, Copenhagen, Denmark; Nathan S Kline Institute, UNITED STATES

## Abstract

**Background:**

Both ^18^F-fluoro-deoxy-glucose (FDG) positron emission tomography (PET), computed tomography (CT) and magnetic resonance imaging (MRI) are routinely used in the evaluation of memory clinic patients. Hybrid PET/MR systems now allow simultaneous PET and MRI imaging within the duration of the PET emission scan.

**Purpose:**

To compare the diagnostic yield of PET/MRI using an abbreviated MR protocol with that of separate PET and CT in a mixed memory clinic population, and the propagated influences on clinical diagnosis and patient management.

**Material and methods:**

Consecutive memory clinic patients (n = 78) undergoing both CT and hybrid FDG PET/MRI scans were identified retrospectively. MRI and CT were separately evaluated for vascular and structural pathology. PET scans were classified according to the presence of neurodegenerative or vascular disease using CT or MRI, respectively, for anatomical guiding. A memory clinic expert assessed the clinical impact of the additional findings and/or change of PET classification achieved by MRI anatomical guiding as compared to CT guiding.

**Results:**

MRI lead to significantly higher Fazekas scores, higher medial temporal and global cortical atrophy scores, and identified more patients with infarcts (28 vs 8, p<0.001) compared to CT. MRI changed PET classification in 13 (17%) patients. Addition of MRI to CT had minor clinical impact in 4/78 (5%) and major clinical impact in 13/78 (17%) of patients.

**Conclusion:**

The study demonstrates the capabilities of PET/MRI systems for routine clinical imaging of memory clinic patients, and that even an abbreviated hybrid PET/MRI protocol provides significant additional information influencing clinical diagnosis and patient management in a substantial fraction of patients when compared to separate PET and CT.

## Introduction

Brain imaging plays a pivotal role in the evaluation of patients with cognitive complaints. Structural imaging using computed tomography (CT) or magnetic resonance imaging (MRI) is recommended in all patients in order to exclude potentially reversible causes of dementia, to demonstrate presence of vascular disease or characteristic patterns of regional atrophy [1]. Magnetic resonance imaging (MRI) is often considered superior to CT in dementia imaging [2], in particular for demonstrating vascular lesions and for identifying additional pathology, e.g. microbleeds and white matter lesions.

Radionuclide studies using ^18^F-fluoro-deoxy-glucose (FDG) positron emission tomography (PET) imaging of glucose metabolism or single photon emission tomography (SPECT) imaging of cerebral perfusion has been shown to provide valuable information of neuronal function and integrity [3], and FDG PET has been shown to be superior to both perfusion SPECT and MRI hippocampal volume measurements for detection of Alzheimer’s disease [4].

A multimodal approach combining FDG PET and MRI providing complementary information may thus be optimal [5], and co-registration with structural imaging is recommended when evaluating FDG PET in order to correctly assess structural correlates of FDG PET abnormalities [6].

Currently, FDG PET can be obtained on standard PET/CT and read with CT acquired separately (or in the same scanning session) or with separately obtained MRI. With the introduction of hybrid PET/MRI systems, the added diagnostic value of MRI may now also be included in a single imaging procedure. Hybrid PET/MRI systems are becoming increasingly available and the potential clinical and research applications have been the subject of a number of recent reviews [7–11]. However, PET/MRI is still a new technology and its potential clinical use is not addressed in current guidelines. Studies on routine clinical use of hybrid FDG PET/MRI in a mixed memory clinic population are sparse, and no previous studies have investigated how PET/MRI compares to PET/CT in terms of diagnostic information including interpretation of FDG PET, clinical diagnosis and patient management.

When implementing the PET/MRI system for clinical routine at our institution, we initially used the system for FDG PET only. In order to complete the scan within the recommended PET duration of 10 minutes [6], the MRI protocol was limited to T2 and 3D T1 weighted MRI not intended for diagnostic purposes, and clinical CT obtained elsewhere was used. These data provide the opportunity to retrospectively investigate the diagnostic yield of a time efficient combined FDG PET/MRI protocol compared to separate PET and CT in clinical routine.

The aims of the present study were to assess the clinical value of hybrid PET/MRI in clinical routine in memory clinic patients, and specifically to compare the diagnostic yield of an abbreviated hybrid PET/MRI protocol with that of separate PET and standard CT, and to compare the propagated influences on interpretation of FDG PET, clinical diagnosis and patient management.

## Material and methods

FDG PET/MRI studies performed in patients referred from the Memory Clinic at Rigshospitalet between January 2013 and May 2014 were retrospectively identified. All patients attended the memory clinic for evaluation of suspected dementia due to cognitive dysfunction reported by the patient, caregivers or health professionals. At the discretion of the memory clinic physician FDG PET was included to in the work-up to support or exclude neurodegenerative disease as a cause of cognitive dysfunction, and patients without contraindication to MRI were eligible to imaging on PET/MRI. Patients with a diagnostic quality non-enhanced cerebral CT performed within ±3 months of the PET/MRI were included in the present retrospective analysis. CT studies of non-diagnostic quality were excluded. A total of 78 patients (32 males/46 females with a mean age of 76 [range 50–89] years) met the inclusion criteria Average time between CT and PET/MRI was 16 (range 0–88) days.

Due to the retrospective design of the study, approval of the region ethics committee was not required. Use of patient data was approved by the Danish Patient Safety Authority (ref. 3-3013-1513/1).

### Imaging procedures

PET/MRI imaging was done following 6 hour fasting. The patient was equipped with earplugs and had eyes covered, after which approximately 200 MBq F-18 FDG was injected intravenously. Forty minutes after tracer injection a single-bed 10 min simultaneous PET/MRI acquisition was performed on a Siemens Biograph mMR 3T PET/MRI system (Siemens Healthcare, Erlangen, Germany). Spatial resolution of the system is approx. 5 mm (FWHM, 10 cm from center of FOV) [12]. MRI acquisition started at the same time as the PET emission scan commenced, and all MRI imaging was completed within the duration of the PET emission scan (overview and details of protocols are provided in supporting information S1 Fig and S1 Table) PET images were reconstructed into a 344x344 matrix (voxel-size 0.8.x0.8x2 mm^3^, zoom 2.5) using 3D OP-OSEM (4 iterations, 21 subset) and applying a 3 mm Gaussian filter in order to match image quality on our clinical PET/CT systems.

Immediately prior to or after PET/MRI imaging a non-diagnostic low dose CT (120 kV, 30 mAs, 5 mm slice width) was obtained on a clinical PET/CT system (Siemens Biograph, Siemens, Erlangen, Germany). The low dose CT was solely used for attenuation correction of PET using a previously described off-line approach [13] by which the low dose CT is co-registered to the 3D T1 MRI from PET/MRI and generating a CT based mu-map in PET/MRI space (as 3D T1 and PET are acquired simultaneously) that can be applied to PET reconstruction on the scanner.

The abbreviated MRI protocol included axial T2-weighted turbo spin echo (TR/TE 6000/106 ms, flip angle 150°, 48 slices, voxel size 0.7×067×3.0 mm^3^), and 3D T1-weighted T1-weighted magnetization-prepared rapid acquisition gradient-echo (MPRAGE, TR/TE/TI 1900/2.44/900 ms, flip angle 9°, 192 slices, voxel size 1.0×1.0×1.0 mm^3^) sequences.

Available CT studies were performed at different hospitals on a variety of scanners using a range of imaging protocols optimized to each scanner type according to quality standards of the respective hospitals. All included standard 5 mm reconstructed images in axial, sagittal and coronal planes. Only studies of diagnostic quality were included.

### Image reading

CT and MRI studies were read independently in a random order by the same radiologist (NK) blinded to all patient information. CT studies were read first, and MRI studies were read at least 1 month after completion of the CT readings to avoid recall bias. Studies were scored for global cortical atrophy (GCA) [14], medial temporal lobe atrophy (MTA) [15], and white matter lesions (Fazekas scale) [16,17], and also infarcts and other visible pathology (e.g. hygromas, tumors, etc.) were noted. Vascular lesions were evaluated according to International Society for Vascular Behavioral and Cognitive Disorders (VASCOG) radiological criteria for mild or major vascular cognitive disorder [18].

Each PET study was read by one of two nuclear medicine physicians experienced with brain FDG PET (OH and LM), first with CT (referred to as PET/CT) and again with MRI at least one month apart. Images were read in randomized order blinded to all clinical information except for the corresponding radiology scoring sheet. For each patient, the two readings were performed by the same reader. FDG PET was read co-registered and superimposed to CT or MRI using a standard clinical work station (MI Neurology, SyngoVia, Siemens HealthCare, Erlangen, Germany) also providing access to statistical comparison with age-matched FDG PET normal databases (applying cerebellar normalization) provided by the software vendor. PET scans were classified into five main diagnostic groups: “Normal” or either “Neurodegenerative”, “Vascular”, “Both vascular and neurodegenerative”, or “Other abnormal”.

The degree to which the PET changes could be attributed to the structural or vascular abnormalities was assessed on a scale from 1 (not at all) to 5 (completely). Also, the reader indicated the level of subjective diagnostic confidence on a 10 cm visual analogue scale (VAS).

In cases where PET classification changed from PET/CT to PET/MRI it was assessed if the change of interpretation was supported by additional findings on MRI (concordant change). If the change was considered to be discordant, both scans were re-evaluated by both readers in conjunction, and it was by consensus assessed if the change was considered to reflect reproducible change of interpretation (n = 5) or random intra-observer variation (n = 6). In the latter case the scans were re-classified according to a consensus decision.

### Clinical assessment

Results of the readings and classifications were presented to a senior memory clinic neurologist (SH). The clinical diagnosis assigned after standard diagnostic work-up was based on clinical and neuropsychological evaluation, imaging (also imaging not included in the present analysis) and laboratory results including cerebrospinal fluid (CSF) analysis. Each case was classified into one of five main diagnostic groups according to the presence and cause of cognitive dysfunction: “Normal”, “Neurodegenerative”, “Mixed”, “Vascular”, or “Other abnormal”. In each case the clinician assessed if the additional information from MRI or re-classification of FDG PET was considered to change patient management. Minor change entailed changes in diagnosis, but without change in treatment or medication. Major change entailed changes in treatment or medication. Changes in treatment of cerebrovascular disease were estimated according to current guidelines [19].

### Statistics

Continuous data are presented as median (range). P-values for paired comparison of continuous and ordinal data were calculated using the Wilcoxon signed rank test. For 2x2 contingency tables Fischer’s exact test was applied. All statistics were done using STATA version 13 (StataCorp LLC, College Station, Tx).

## Results

**Fig 1:**
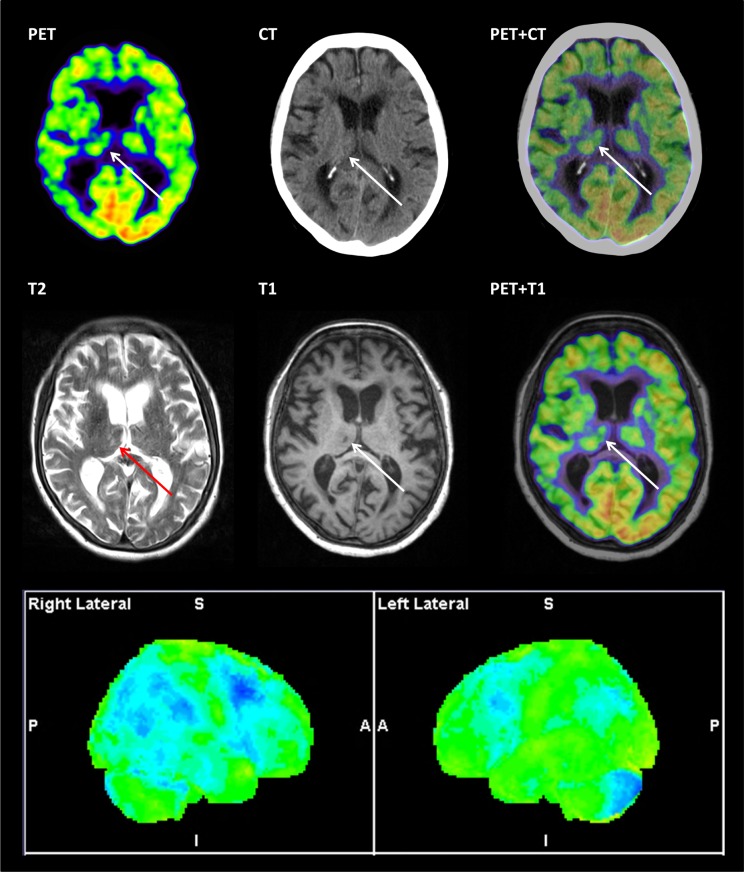
Major clinical impact. Upper rows show corresponding axial slices from CT, T2 and T1 MRI, and fused PET+CT and PET+T1. The two scans were performed 7 days apart. MRI shows a lacunar infarct in the right thalamus not visible on CT. Both MRI and CT were scored as Fazekas 2. Fused FDG PET and T1 MRI show a small metabolic defect in the right thalamic infarct (arrows). Statistical surface projections (lower row) show frontal and parietotemporal hypometabolism more pronounced in the right cerebral hemisphere and also lower uptake in the left cerebellar hemisphere. PET classification changed from neurodegenerative + vascular disease (white matter lesions) to predominantly vascular disease (white matter lesions + strategic infarct). Clinical diagnosis was changed from neurodegenerative to mixed and the finding was considered to constitute major impact (indication for platelet inhibitory drug).

**Fig 2:**
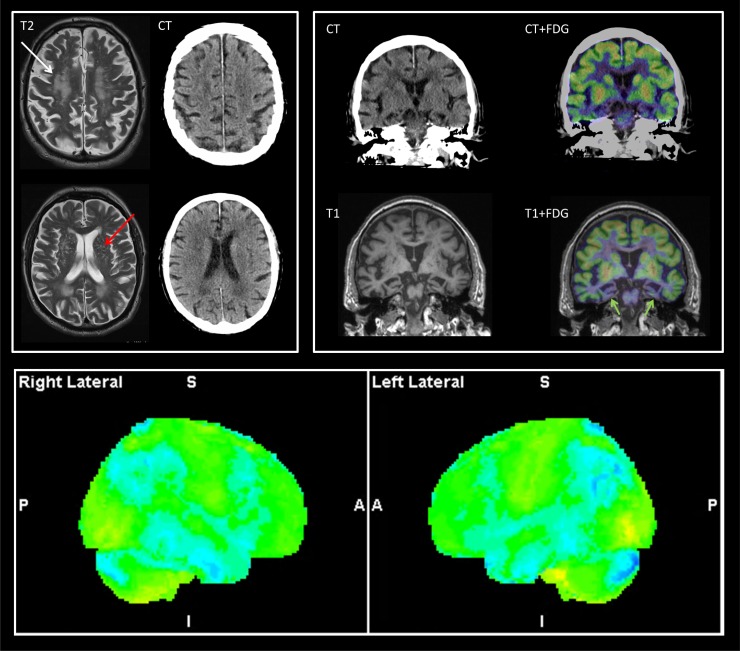
Minor clinical impact. CT and MRI performed 10 days apart. T2 MRI shows more pronounced white matter lesions (white arrow) compared to CT (Fazekas score 3 vs 2) and demonstrates also widening of perivascular spaces in the basal ganglia (red arrow) often considered a sign of vascular disease (état criblé). T1 MRI showed more pronounced atrophy of mesial temporal lobe compared to CT (upper right panel) scored as MTA 3 vs 2 on CT. FDG PET shows parietotemporal hypometabolism with involvement also of mesial temporal structures (green arrows, upper right panel) suggestive of Alzheimer’s disease. With MRI, classification of PET changed from neurodegenerative disease to neurodegenerative + vascular disease and the clinical diagnosis changed from neurodegenerative disease to mixed dementia. As the change did not prompt change of therapy, but only diagnosis, the change was classified as minor impact.

Patient characteristics are summarized in Table 1. Two examples of PET/MR and PET/CT imaging are presented in Figs 1 and 2. In both cases additional vascular pathology was identified on MRI compared to CT that in turn altered the interpretation of FDG PET.

**Table 1:** Demographic and background information.

MMSE, median (range)	24.2±4.0 [15;30]†
**Clinical diagnosis, (%)**	
**Neurodegenerative [AD/eAD/DLB/FTD/other]**	43 (55%) [35/2/4/1/1]
**VaD**	6 (8%)
**Mixed**	7 (9%)
**Other abnormal**	13 (17%)
**Normal**	9 (12%)

†MMSE (n = 74) or RUDAS (n = 2) score at the time of referral.

Abbreviations: AD = Alzheimer’s disease, eAD = early or possible AD, DLB = dementia with Lewy bodies, FTD = fronto-temporal dementia, VaD = vascular dementia, Mixed = mixed vascular dementias and Alzheimer’s disease, MMSE = mini mental state examination, RUDAS = Rowland Universal Dementia Assessment Scale.

### Structural scores and other pathology

MRI identified significantly more infarcts compared to CT, demonstrating one or more infarcts in 28 patients compared to nine patients using CT (p<0.001). Fig 1 shows example of a thalamic lacunar infarct on MRI not visible on CT. In seven of the nine patients with infarcts on CT, MRI demonstrated additional infarcts. Using CT four scans fulfilled VASCOG criteria for mild cognitive disorder and two scans fulfilled criteria for major cognitive disorder. For MRI criteria for mild and major cognitive disorder were fulfilled in two and 14 scans, respectively. The frequency of patients with additional vascular pathology on MRI was not different between those with CT performed before vs after MRI (17/48 vs 8/25, p = 0.61).

MRI yielded significantly higher Fazekas score, GCA and MTA scores compared to CT (Table 2). An example of higher Fazekas on MRI scores is shown in Fig 2.

**Table 2:** Radiology and PET reader scores.

	CT	MRI	p-value
**Radiological scores**			
GCA (0/1/2/3)	12/22/35/9	6/21/38/13	<0.001
MTA (0/1/2/3/4)	18/19/25/14/2	13/21/27/14/3	0.020
Fazekas score (0/1/2/3)	48/10/18/2	39/14/22/3	<0.001
**PET reader scores**			
Vascular contribution (1/2/3/4/5)	55/18/3/2/0	54/9//10/4/1	0.036
Structural contribution	15/23/23/12/5	15/23/18/19/3	0.544
VAS subj. confidence, mm	82±13	88±11	<0.001

Abbreviations: GCA = global cortical atrophy score, MTA = mesial temporal atrophy score, VAS = visual analog scale of reader confidence.

### Influence on interpretation of FDG PET

Diagnostic classification of PET/CT and PET/MRI are summarized in Table 3. Of the 78 patients, 13 (17%) were classified at least partially different on PET/MRI versus PET/CT. The most common change of interpretation was that among 46 patients classified as neurodegenerative on PET/CT eight patients (17%) were re-classified to neurodegenerative+vascular, and in 8/13 patients reclassified, the change in classification was concordant with additional vascular pathology found on MRI compared to CT (see examples in Figs 1 and 2) In the five cases with non-concordant change, the change in interpretation was related to more conspicuous white matter lesions (n = 3) or atrophy (n = 2) on MRI compared to CT.

**Table 3:** Changes in main PET classification from PET/CT to PET/MRI.

PET/MRI	PET/CT					
Classification	Normal	ND	Vascular	ND + vascular	Other	Total
Normal	19	0	0	0	0	19
ND	*2*	36	0	0	0	38
Vascular	*1*	0	2	*1*	0	4
ND+vascular	0	*8*	0	2	0	10
Other	0	*1*	0	0	6	7
Total	22	45	2	3	6	78

Abbreviations: ND = neurodegenerative. Number of patients in whom PET interpretation changed from PET/CT (columns) to PET/MR (rows) is highlighted in italic.

The scores for vascular contribution for FDG classification were significantly higher for MRI compared to CT. There was a significant increase in subjective diagnostic confidence from PET/CT to PET/MRI (Table 2).

### Influence on clinical assessment and treatment

Influence of PET/MRI on clinical diagnosis is summarized in Table 4. In accordance with the change of PET interpretation above, the most frequent change was from neurodegenerative disease on PET/CT to mixed or vascular on PET/MR (total of 10 of 45 patients) In seven of the 13 cases reclassified on PET/MRI, the reclassification had minor impact in two patients and major impact in five patients (i.e. indication for the platelet inhibitory drug clopidogrel). In all these patients more vascular pathology was demonstrated on MRI compared to CT. Figs 1 and 2 shows examples of major and minor impact respectively.

**Table 4:** Changes in main clinical diagnosis.

Revised diagnosis	Clinical diagnosis					
	Normal	ND	Vascular	Mixed	Other	Total
Normal	9	0	0	0	0	9
ND	0	35	0	0	0	35
Vascular	0	*1*	6	*1*	*1*	9
Mixed	0	*9*	0	4	0	13
Other	0	0	0	0	12	12
Total	9	45	6	5	13	78

ND = neurodegenerative. Number of patients in whom diagnosis changed from clinical diagnosis assigned after standard work-up (columns) to revised diagnosis (row) with PET/MR is highlighted in italic.

Among those not reclassified on PET/MRI, MRI showed infarcts in 14 patients in whom CT had not shown any infarcts, and identified additional infarcts in another 5 patients. Of these 19 patients, the findings were considered to have major clinical impact in 8 patients (indication for clopidogrel) and minor impact in 2 patients (change from AD to mixed).

In total, the additional findings and change of interpretation of PET/MR compared to PET/CT were considered to influence patient diagnosis or management in 17 (22%) of the patients, having major impact in 13 (17%) and minor impact in 4 (5%) patients.

## Discussion

Previous studies reporting use of PET/MRI system in memory clinic patients have in general included relatively few patients and have focused on feasibility [20–22] and on the influence of attenuation correction [13,23,24]. The present study is the first to report on routine clinical use of hybrid PET/MRI in a large memory clinic population, and also the first to compare the diagnostic yield of MRI and CT in hybrid FDG PET imaging in these patients. The main findings are that hybrid FDG PET/MRI imaging using an abbreviated MRI protocol revealed more vascular pathology in 35% of patients, induced a change of the interpretation of FDG PET in 17% of patients, and was considered to influence patient management in 22% of patients.

It is well established that MRI is more sensitive for detection of vascular pathology compared to CT, and is often also considered superior to CT in dementia imaging. However, as pointed out by others [2,25] this notion is largely based on older studies involving single slice CT and lower field MRI. A study comparing 64 slice CT with 1.5 T MRI found MRI to be more sensitive for detecting primarily low grade white matter lesions, but otherwise showed no systematic differences between CT and MRI for GCA, MTA and Fazekas score [25]. In the present study MRI did not only identify more infarcts, but MR also influenced the interpretation of PET as a vascular contribution to hypometabolism was more often identified. In all cases with influence on patient management, the change of management was related to additional vascular pathology on MRI.

Also atrophy scores were higher using MRI. GCA evaluation was done using T1 and T2 and not T2 fluid-attenuated inversion recovery (FLAIR), which could potentially overestimate the cortical atrophy [26,27]. We also found significantly higher MTA score using high field MRI than CT. This observation in agreement with a recent study showing that 256 slice CT underestimated MTA score compared 3T MRI [28]. Our findings thus add to the evidence supporting MRI as the preferred structural imaging modality in dementia work-up.

To our knowledge, no previous studies have investigated the influence of structural imaging modality on PET reading. The higher subjective confidence of FDG PET reading could be related to a more confident assessment of the structural correlates due to higher image contrast and resolution in T1 MPRAGE compared to CT. To minimize the influence of random intra-observer variation, we performed consensus reading in those reclassified without significant additional findings on MRI. On re-assessment, 6 of 11 cases were considered to reflect random variation and the remaining to reflect reproducible change of interpretation. Also, there appears to be quite clear systematic differences between CT and MRI and subsequent PET interpretation (primarily additional vascular contributions) that are unlikely to be the result of random variation.

The sequences included in the abbreviated PET/MR protocol was restricted to T1 and T2 weighted sequence in order to complete the study within the 10 minute duration of the PET study. The 3D T1 was included for anatomical guidance and assessment of atrophy, and axial T2 was chosen for its high sensitivity to ischemic pathology, and may also show edema, tumors and other pathology. The 3D T1 was also required for co-registration with low-dose CT used for attenuation correction.

The protocol did not include T2* or susceptibility weighted (SWI) sequences which made it impossible to asses prior hemorrhages and microbleeds, and possible cerebral amyloid angiopathy. In the study population four patients had regular MRI performed within ±3 months of the PET/MR with abbreviated MR protocol. In one case microbleeds were reported, but otherwise no additional diagnostic information was provided compared to abbreviated MR from PET/MRI. From June 2014 we employed a fully diagnostic MR protocol including also T2* weighted, diffusion weighted imaging (DWI) and T2 fluid-attenuated inversion recovery (FLAIR) sequences in the standard PET/MR protocol, and MRI imaging was now read and reported by an experienced neuroradiologist. To assess the frequency of findings not detectable on sequences included in the abbreviated protocol we reviewed diagnostic reports from 100 consecutive scans with full MRI. Findings on T2* were reported in 18 scans (of which 9 cases were considered unspecific age related microbleeds and 3 cases with possible amyloid angiopathy), but no relevant findings were made on DWI. Findings on T2* in these 18 patients were considered to have impact on treatment in one patient (major impact), and supported an uncertain diagnosis in another patient (minor impact). This review indicate that findings on T2* MRI are frequent, although not always clinically important, whereas DWI did not provide clinically significant information. In younger patients or patients with rapidly progressing dementia DWI should be considered in order to evaluate for vasculitis or Creutzfeldt-Jakob disease. Patients in these categories are infrequent in the population referred from a tertiary dedicated memory clinic, and will usually already have had comprehensive MRI performed before FDG PET imaging is required.

We have now included T2* and T2 FLAIR in the standard fully diagnostic PET/MRI dementia protocol, whereas DWI is only performed in younger patients and in patients with rapidly progressive disease. Using this dementia protocol (see S1 Fig and S1 Table for overview and details of sequence parameters), we have adopted FDG PET/MRI into clinical routine and are currently performing approximately 300 examinations per year.

In spite of the above-mentioned limitations of the MRI protocol we have shown that even using only axial T2 and 3D T1 sequences not prolonging the scan time, significant additional findings are made that in turn influences diagnostic classification and patient management.

There are some limitations as to the comparison of CT and MRI. MRI was not performed at the same time as CT, and lesions could in some cases have developed or progressed within the time interval between the two exams. Still, the frequency of additional infarcts was not related to whether MRI was performed before or after CT. In some cases additional finding made on MRI may in retrospect also be identified on CT, but in order to reflect clinical routine, we have not revised the CT readings. Furthermore, CT was performed on a variety of scanners. It is possible that uniform high-quality imaging from a state-of-the-art CT scanner in all patients would have yielded a higher diagnostic sensitivity and subjective confidence for PET/CT. However, we believe that the variability in CT reflects the present standards in clinical routine.

Due to the retrospective design of the study, it may be difficult to accurately assess the clinical impact of PET/MR imaging. The impact on management is most likely underestimated as we compared to the diagnosis and treatment based on the full clinical work-up including additional imaging and other information not included in the present analysis. Infarcts only found on MRI were known from prior imaging in three cases and classified as no impact (n = 2) or minor impact (n = 1), but would have major clinical impact in a prospective design. It should be noted though, that although additional infarcts identified on MRI in many cases prompted change of therapy and thus constituted major impact, the findings in some cases did not change interpretation of PET or the clinical diagnosis as to the cause of cognitive impairment, and should probably be regarded as incidental findings and not a cause of cognitive dysfunction.

Another limitation is that diagnostic accuracy cannot be assessed. Lacking a diagnostic gold standard long term follow-up would have been of value in order to revise or confirm the clinical diagnosis. However, as patients attended a tertiary university memory clinic, the vast majority of patients returned to their local hospital or general practitioner, and no systematic follow-up is possible. The study is thus limited to the diagnostic information yielded and immediate influence on patient management.

It may be debated if a clinical impact in approximately 22% of patients justifies routine combined MRI and PET imaging in all patients. Currently we are still relying on separate low-dose CT for attenuation correction and a longer combined protocol of approx. 17 minutes in order to include all standard MRI sequences. At the time of the study only MRI based attenuation correction using the Dixon sequence was available which as demonstrated previously is associated with a strong radial bias compared to low-dose CT [13] that in our opinion is not acceptable for clinical use. Recent developments in MRI based attenuation correction [29] combined with machine learning [30] has greatly improved accuracy of MRI based attenuation correction, and may supersede the need for low-dose CT, although mainly clinically validated in tumor imaging [29,31]. At our department, we are currently in the process of validating such an approach for clinical imaging. Use of synthetic MRI protocols for standard T1 and T2 weighted sequences [32] may reduce total acquisition time to the duration of the PET scan and thus improve the cost-effectiveness of FDG PET/MRI in clinical use. Using hybrid PET/MRI systems may improve both patient/caregiver and referrer convenience in dementia imaging [30], but is in terms of diagnostic information provided and image quality comparable to sequential imaging with stand-alone MRI and PET systems that remain a reliable and cost-effective alternative. The results of the present study thus highlight the importance of integrating MRI information in the reading of the PET scan which is reliably ensured by hybrid imaging.

In conclusion, the study demonstrates the application of hybrid PET/MR systems in routine clinical brain imaging in memory clinic patients, and also shows the superiority of an abbreviated MRI protocol compared to CT in terms of vascular pathology leading to clinically important changes of patient management in a substantial fraction of patients. We believe that the study thus highlights the general capabilities of hybrid PET/MRI systems beyond research applications for improved diagnostic accuracy in clinical routine brain imaging. The use of hybrid systems limiting the number of scanning procedures is especially important in a fragile elderly population.

## Supporting information

S1 Table Details of PET and MR imaging parameters. (DOCX)

S1 Fig PET-MR protocols. (DOCX)
