# Supplementary material for: Hybrid FDG PET/MRI vs. FDG PET and CT in patients with suspected dementia – A comparison of diagnostic yield and propagated influence on clinical diagnosis and patient management
Source: PLoS One. 2019 May 2;14(5):e0216409. doi: 10.1371/journal.pone.0216409 (PMC6497285; doi:10.1371/journal.pone.0216409)
Supplement: S1 Table — (DOCX) [file pone.0216409.s001.docx]

**Supporting information S1 Table. Details of PET and MR imaging parameters.**

|  | Abbreviated protocol | Current full protocol |
| --- | --- | --- |
| 3D T1 | Sagittal, 192 slices  GRAPPA 2  Flipangle 9 deg  TR/TE/TI 1900/2.44/900 ms  FOV /Matrix: 250 mm/256x256  Voxel size 1x1x1 mm  Time acq.: 5:04 | Sagittal, 192 slices  GRAPPA 2  Flipangle 9 deg  TR/TE/TI 1900/2.44/900 ms  FOV /Matrix: 250 mm/256x256  Voxel size 1x1x1 mm3  Time acq.: 5:04 |
| T2 | Transverse TSE 48 slices  Flipangle 150 deg  TR/TE 6000/105 ms  FOV /Matrix: 250 mm/358x448  Voxel size 0.7x0.6x3 mm  Time acq.: 4:08 | TRA (BLADE)  Flipangle 90 deg  TR/TE 5550/117 ms  FOV /Matrix: 230 mm/256x256  Voxel size 0.7x0.7x5 mm  Time acq.: 3:21 |
| Axial T2 FLAIR | Not included | Transverse,  GRAPPA 3  TR/TE/TI 9000/58/2500 ms  FOV /Matrix: 230 mm/320x320  Voxel size 0.4x0.4x5 mm  Time acq.: 2:26 |
| T2* | Not included | Transverse, 35 slices  GRAPPA 2  TR/TE/TI 620/19.9 ms  FOV /Matrix: 230 mm/256x256  Voxel size 0.4x0.4x5 mm  Time acq.: 4:16 |
| DWI RESOLVE | Not included | (optional)  Transverse, 25 slices  TR/TE1/TE2 5600/63/101 ms  FOV /Matrix: 220 mm/192x192  Voxel size 1.1x1.1x4 mm  b-vaules 0/800 s/mm^2^  Time acq.: 4:19 |
| PET | 10 min list mode acquisition  FOV/matrix/zoom 58.8 cm/, 344x344/2.5  Voxel size 0.8x0.8x2 mm  3D OSEM with 3 mm Gassian filter | 10 min list mode acquisition  FOV/matrix/zoom 58.8 cm/, 344x344/2.5  Voxel size 0.8x0.8x2 mm  3D OSEM with 3 mm Gassian filter |
