# Supplementary figures and images for: Hybrid FDG PET/MRI vs. FDG PET and CT in patients with suspected dementia – A comparison of diagnostic yield and propagated influence on clinical diagnosis and patient management
Source: PLoS One. 2019 May 2;14(5):e0216409. doi: 10.1371/journal.pone.0216409 (PMC6497285; doi:10.1371/journal.pone.0216409)

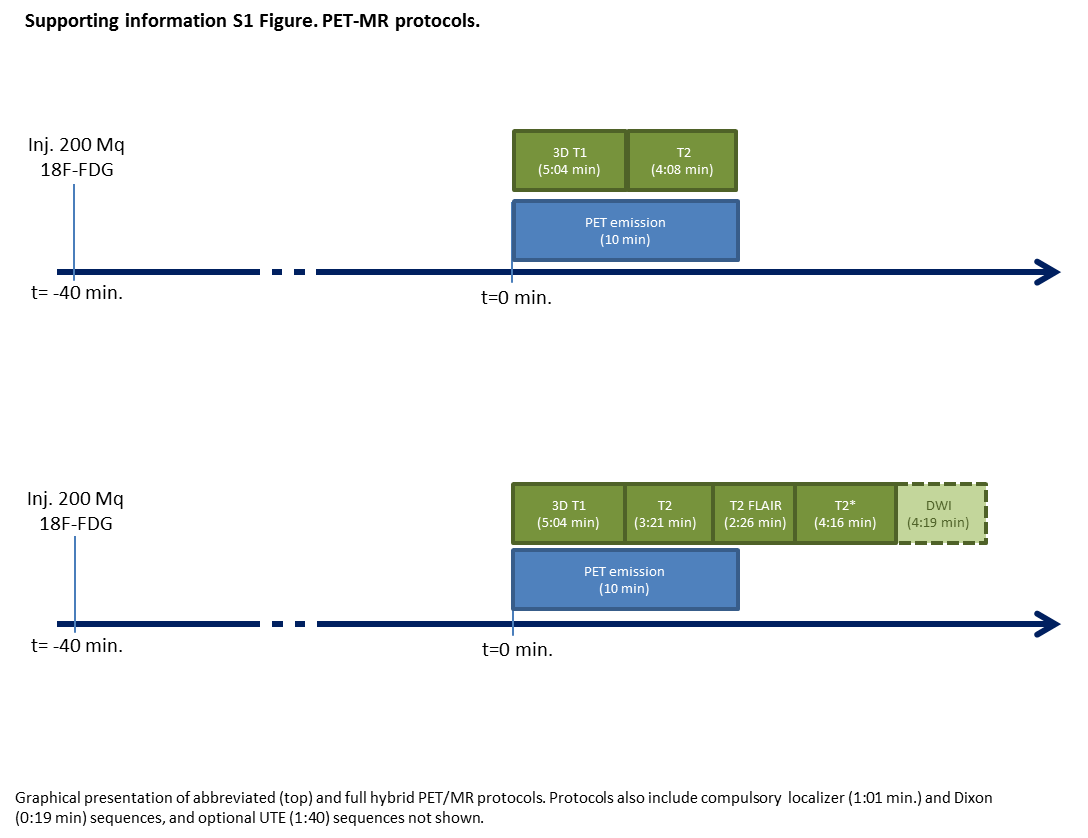

Supplement: S1 Fig — (DOCX) [file pone.0216409.s002.docx]
